# Synthesis of a Novel Unexpected Cu(II)–Thiazolidine Complex—X-ray Structure, Hirshfeld Surface Analysis, and Biological Studies

**DOI:** 10.3390/molecules27144583

**Published:** 2022-07-18

**Authors:** Mezna Saleh Altowyan, Samar M. S. M. Khalil, Dhuha Al-Wahaib, Assem Barakat, Saied M. Soliman, Ali Eldissouky Ali, Hemmat A. Elbadawy

**Affiliations:** 1 Department of Chemistry, College of Science, Princess Nourah bint Abdulrahman University, P.O. Box 84428, Riyadh 11671, Saudi Arabia; msaltowyan@pnu.edu.sa; 2 Chemistry Department, Faculty of Science, Alexandria University, P.O. Box 426, Alexandria 21321, Egypt; samar.mohamed_pg@alexu.edu.eg (S.M.S.M.K.); saied1soliman@yahoo.com (S.M.S.); 3 Chemistry Department, Faculty of Science, Kuwait University, Kuwait City 13060, Kuwait; d.alwaheeb@ku.edu.kw; 4 Department of Chemistry, College of Science, King Saud University, P.O. Box 2455, Riyadh 11451, Saudi Arabia; ambarakat@ksu.edu.sa

**Keywords:** Cu(II)–thiazolidine, hydrazone, XPS, Hirshfeld, antibacterial, colon carcinoma

## Abstract

An unexpected trinuclear Cu(II)–thiazolidine complex has been synthesized by mixing CuCl_2_·2H_2_O with the Schiff base ligand, 1-(((4,5-dihydrothiazol-2-yl)ethylidene)hydrazono)methyl)phenol **L**, in ethanol. Unexpectedly, the reaction proceeded via the hydrolysis of the Schiff base **L**, followed by cyclization to afford 3-methyl-5,6-dihydrothiazolo[3,2-*c*][1,2,3]triazole (**L^a^**), then complexation with the Cu(II) salt, forming the trinuclear **[Cu_3_(L^a^)_4_(Cl)_6_]** complex. The complex was characterized by means of FTIR spectra, elemental analysis, and X-ray crystallography. In the trinuclear **[Cu_3_(L^a^)_4_(Cl)_6_]** complex, there are two crystallographically independent hexa- and penta-coordinated Cu(II) sites, where the thiazolidine ligand **L^a^** units act as a monodentate ligand and a linker between the Cu(II) centers. The crystal packing of the **[Cu_3_(L^a^)_4_(Cl)_6_]** complex is primarily affected by the weak non-covalent C-H∙∙∙Cl interactions. In accordance with Hirshfeld surface analysis, the Cl∙∙∙H, H∙∙∙H, S∙∙∙H, and N∙∙∙H percentages are 31.9%, 27.2%, 13.5%, and 9.9%, respectively. X-ray photoelectron spectroscopy confirmed the oxidation state of copper as Cu(II), as well as the presence of two different coordination environments around copper centers. The complex showed interesting antibacterial activity against the Gram-positive bacteria *S. subtilis*, with MIC = 9.7 µg/mL compared to MIC = 4.8 µg/mL for the control, gentamycin. Moreover, the Cu(II) complex showed an equal MIC (312.5 µg/mL) against *C. albicans* compared to ketoconazole. It also exhibits a very promising inhibitory activity against colon carcinoma (IC_50_ = 3.75 ± 0.43 µg/mL).

## 1. Introduction

Copper is one of the most widely used metals [1,2], with a very low toxic limit [3,4]. It acts as a vital micronutrient and plays various functions in biological systems. Recently, copper compounds have been recommended as therapeutic agents against cancer [5], microbial diseases [6,7,8,9], chronic lung inflammation [10], influenza A [11], neurodegenerative diseases such as Alzheimer’s, Parkinson’s and prion diseases, in addition to disorders related to copper homeostasis, such as Wilson’s and Menkes disorders [12,13,14,15]. Moreover, the copper coordination complexes were considered as promising multi-functional materials due to their success in photophysical and photoelectrochemical applications. These complexes were considered as better alternatives to other transition metal complexes (Pt(II), Ir(III) and Ru(II)) which are expensive, toxic, and of low abundance [16,17]. In contrast, the copper coordination complexes are characterized by their ease of preparation, and they also exhibit low toxicity. Moreover, coordination complexes of copper have been extensively studied and implemented as photosensitizers, triplet emitters, and catalysts in a variety of photochemical applications [18].

Schiff bases have become an encouraging choice as functional ligands in coordination chemistry because of their synthetic flexibility, structural diversity, varied denticity [19], sensitivity, and selectivity towards metal ions. Predominantly, the hydrazone-containing azomethine group (-C=N-N=C-) has been considered as powerful ligands due to their ability to stabilize various metal ions, with different oxidation states producing a variety of molecular designs and geometries [20,21,22,23,24,25,26,27,28]. In addition, the aryl hydrazone metal complexes were found to have interesting electrical and magnetic properties, and were utilized as novel heterogeneous catalysts in redox, chemical, and photochemical reactions in several industrial applications [29,30,31].

**Figure 1:**
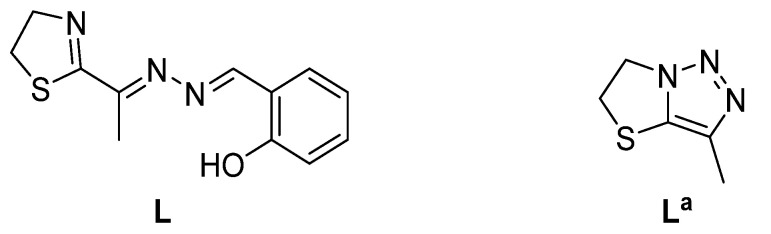
Structure of the presented ligands 1-(((4,5-dihydrothiazol-2-yl)ethylidene) hydrazone)methyl)phenol, **L**, and 3-methyl-5,6-dihydrothiazolo[3,2-c][1,2,3]triazole, **L^a^**.

On the other hand, the thiazolidine nucleus is well-known in the field of pharmaceutical chemistry. This group of compounds has played a crucial role in organic, bio-organic, and medicinal chemistry [32]. This is because most of the antimicrobial substances, such as penicillin, cephalosporin, narcodicins, and thienamycins, were synthesized from thiazolidines [33,34]. Various pharmacological activities, including antiproliferative, hepatoprotective, antidiabetic, antihypertensive, platelet activating factor antagonist, calcium antagonist, anti-inflammatory, antipyretic, analgesic, anthelmintic, antitubular, antiviral, anticancer, mucolytic, and anti-HIV activities, are associated with compounds containing a thiazolidine nucleus [32]. In this work, the reaction of CuCl_2_·2H_2_O with the hydrazone type ligand, 1-(((4,5-dihydrothiazol-2-yl)ethylidene)hydrazone)methyl)phenol (**L**; Figure 1) in ethanol is presented. The structure of the resulting Cu(II)–thiazolidine complex as an unexpected product was investigated using different spectroscopic techniques and single crystal X-ray analysis. Its antimicrobial, antioxidant, and anticancer activities were also investigated.

## 2. Results and Discussion

### 2.1. Chemistry and Characterizations

**Scheme 1:**
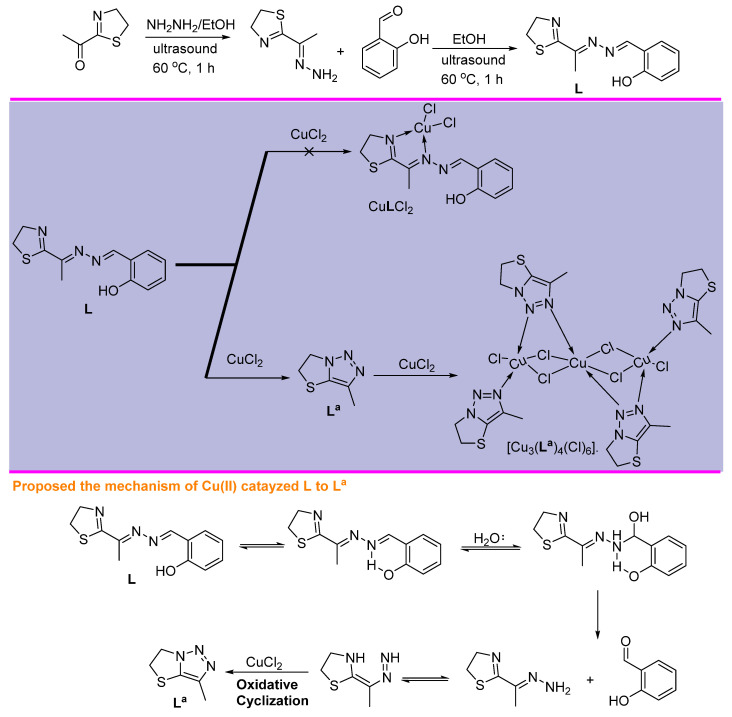
Synthesis of the unexpected **[Cu_3_(L^a^)_4_(Cl)_6_]** complex.

The designed ligand **L** was synthesized according to the method depicted in Scheme 1. The acetyl derivative reacted with hydrazine, followed by reaction with salicylaldehyde to give the corresponding Schiff base **L**. The reaction of CuCl_2_·2H_2_O with 2-(1-(4,5-dihydrothiazol-2-yl)ethylidene)hydrazone)methyl)phenol **L** did not proceed to the formation of the corresponding Cu(II)–hydrazone complex. Unexpectedly, the hydrazone ligand underwent oxidative hydrolysis, followed by cyclization, affording the corresponding thiazolidine **L^a^**, which proceeded to complexation with the Cu(II) ion, affording the trinuclear **[Cu_3_(L^a^)_4_(Cl)_6_]** complex (Scheme 1). Spectral characterizations of the synthesized compounds are presented in Figures S1–S6 (Supplementary Materials). The structure of the novel Cu(II) complex was confirmed using elemental analysis, different spectroscopic measurements, and X-ray crystallography. The antimicrobial, anticancer, and antioxidant activities of the **[Cu_3_(L^a^)_4_(Cl)_6_]** complex were also presented.

### 2.2. X-ray Structure Description of **[Cu_3_(L^a^)_4_(Cl)_6_]** Complex

The X-ray crystallographic measurements confirmed the structure of the trinuclear **[Cu_3_(L^a^)_4_(Cl)_6_]** complex, which is formed via Cu(II)-mediated hydrolysis and cyclization of the hydrazone ligand **L** to the corresponding thiazolidine **L^a^**, followed by complexation with the cupric ion. Table 1 lists the crystallographic data of the trinuclear **[Cu_3_(L^a^)_4_(Cl)_6_]** complex.

**Figure 2:**
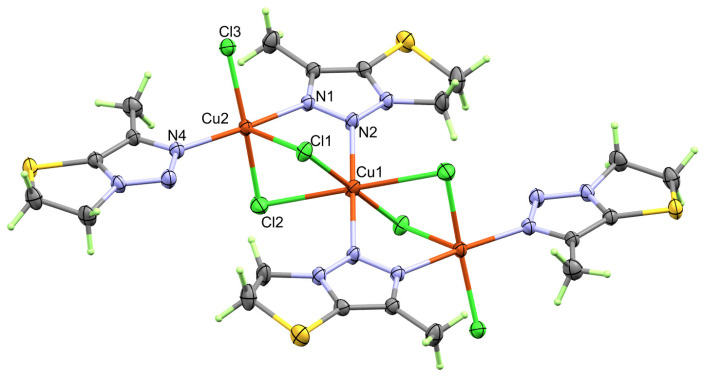
The structure of the trinuclear **[Cu_3_(L^a^)_4_(Cl)_6_]** complex.

The X-ray structure of the trinuclear Cu(II) complex **[Cu_3_(L^a^)_4_(Cl)_6_]** is shown in Figure 2. The results leave no doubt about the oxidative hydrolysis of the Schiff base hydrazone ligand (**L**). The **[Cu_3_(L^a^)_4_(Cl)_6_]** complex crystallized in the triclinic crystal system and *P-1* space group. The unit cell parameters are *a* = 8.1686(3) Å, *b* = 9.4973(4) Å, *c* = 12.7501(5) Å, and *α* = 81.796(2), *β* = 75.291(2)°, *γ* = 65.096(2). The asymmetric unit comprised half of the trinuclear **[Cu_3_(L^a^)_4_(Cl)_6_]** formula, as the complex comprised a center of symmetry located at the Cu(1) site. Hence, there are two different Cu(II) sites in this complex: the central Cu(1), which is hexa-coordinated with four chloride ions, and two nitrogen atoms from two **L^a^** units. Due to symmetry considerations, all the *trans* bonds have an equal length. Hence, there are only three different interactions between the Cu(II) and donor atoms, which are the Cu1-Cl1 (2.2633(10) Å), Cu1-Cl2 (2.8810(11) Å), and Cu1-N2 (2.041(2) Å) bonds, while the other three bonds are symmetry-related and have the same distances, respectively (Table 2). Of course, the bond angles of all the *trans* bonds are 180°, while the angles between the *cis* bonds are in the range of 85.63(3) to 97.12(4) for Cl1-Cu1-Cl2 and Cl1-Cu2-Cl3, respectively. Hence, the structure of the coordination environment could be described as an elongated octahedron, where the two Cl(1) and the two N(2) atoms represent the base, and the two Cl(2) are located as apical. The coordination sphere of the Cu(2) is completely different. The Cu(2) is penta-coordinated, with five coordination interactions. There is one interaction with the terminal Cl(3) atom, in addition to two interactions with the bridged Cl(1) and Cl(2) atoms, which are already coordinated to the Cu(1). The corresponding Cu-Cl distances are 2.2720(12), 2.6178(11), and 2.3140(13) Å, respectively. The coordination environment of the Cu(2) is completed by two interactions with the N(1) and N(4) atoms from two *trans* **L^a^** ligand units. The corresponding Cu-N distances are 2.024(3) and 1.987(3) Å, respectively. The distortion in the CuCl_3_N_2_ coordination sphere of Cu(1) was described based on the criterion of Addison [35]. The largest angles are Cl2-Cu2-Cl3 (β = 171.35°) and N1-Cu2-N4 (α = 166.56°), giving a τ = {(β − α)/60} value of only 0.08. Hence, the coordination geometry is more like to be a distorted square pyramid, where the Cl(1) donor would be regarded as apical. It is worth noting that the structure of this complex comprised two crystallographically independent **L^a^** ligand units, with one of them acting as a terminal ligand, coordinating only the Cu(2) metal site via the significantly short Cu2-N4 bond, and the other **L^a^** unit acting as a connector between the two Cu sites. Hence, this ligand unit and the two Cl1 and Cl2 ions act as a bridging ligand, connecting the Cu(II)-sites leading to the formation of the trinuclear **[Cu_3_(L^a^)_4_(Cl)_6_]** complex.

**Figure 3:**
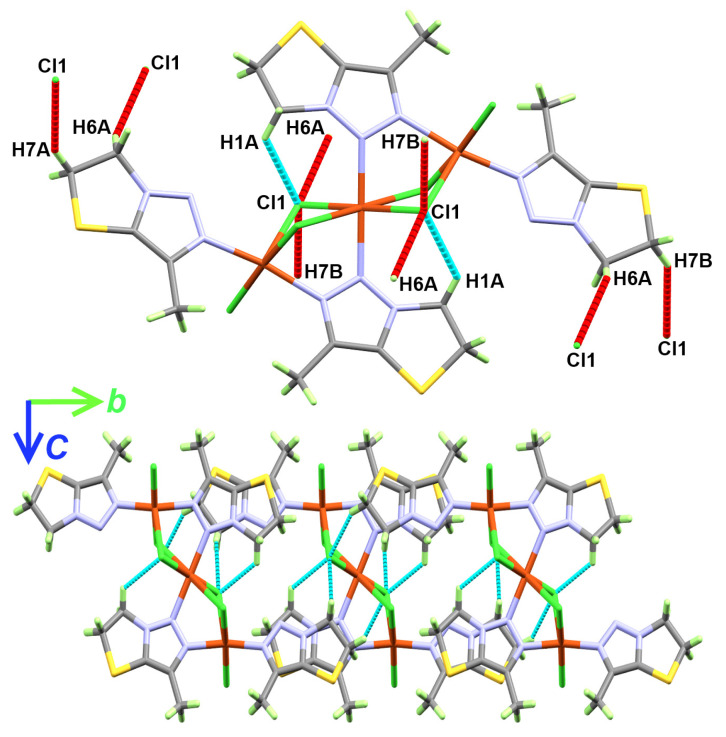
Intra- and intermolecular C-H…O contacts (**upper**) and packing scheme along *bc* plane (**lower**) of the **[Cu_3_(L^a^)_4_(Cl)_6_]** complex.

The structure of **[Cu_3_(L^a^)_4_(Cl)_6_]** is stabilized by an intramolecular C1-H1A∙∙∙Cl1 interaction, with hydrogen–acceptor and donor–acceptor distances of 2.72 and 3.427(5) Å, respectively. Its packing is dominated by the weak non-covalent C-H∙∙∙Cl interactions depicted in Table 3 and shown as a red dotted line in the upper part of Figure 3. A view of the packing through the *bc* plane, showing the complex units connected by the Cl-H∙∙∙O interactions, is presented in the lower part of the same illustration. The donor–acceptor interaction distances range from 3.609(5) Å (C6-H6A∙∙∙Cl1) to 3.692(5) Å for C7-H7B∙∙∙Cl1, respectively.

### 2.3. Hirshfeld Analysis

**Figure 4:**
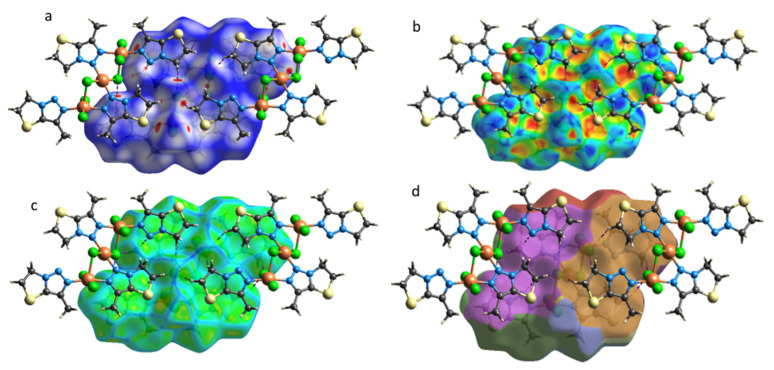
Hirshfeld surfaces mapped with (**a**) *d*_norm_, (**b**) shape index, (**c**) curvedness, and (**d**) fragment patch of the copper complex.

Hirshfeld surface analysis is a powerful tool for visualizing interactions in molecular crystals. Crystal Explorer 17 is used to create the Hirshfeld analysis, analyze the crystal structure of the synthesized complex, and represent intermolecular interactions on that surface [36]. The *d*_norm_ surfaces are mapped in the range of −0.05 to 0.80 Å, while the shape index, curvedness, and fragment patch are mapped over the ranges −1.0 to 1.0 Å, −4.0 to 0.4 Å, and 0 to 15 Å, respectively Figure 4. The *d*_norm_ surface detected the very close intermolecular interactions presented as red spots, indicating short H∙∙∙H, N∙∙∙H, S∙∙∙H, and Cl∙∙∙H interactions. The shape index shows the shape of the surface (concave (−1.0) to convex (+1.0)), while curvedness clarifies the flatness of surface indicated as flat (−4.0) to singular (+0.4). A fragment patch is often used to divide the surfaces into patches, suggesting interactions between neighboring molecules. This mapping color patch allows the identification of the closest neighbor coordination environment of a molecule.

**Figure 5:**
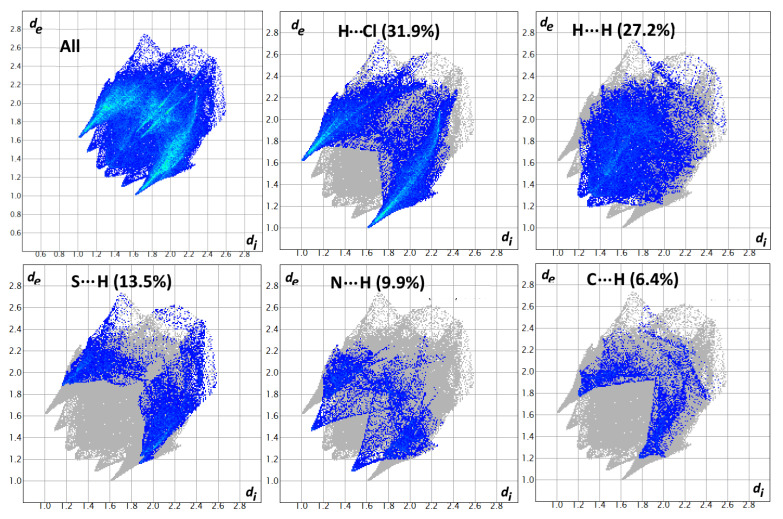
Fingerprint plots for the copper complex, showing percentages of important contact contributions to the total Hirshfeld surface area of the copper molecule.

**Figure 6:**
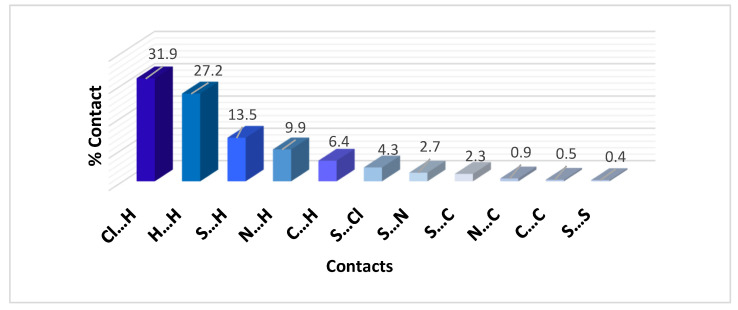
Intermolecular interaction and percentages of all contact contributions to the total Hirshfeld surface analysis.

The fingerprint plots are demonstrated in Figure 5. The complementary regions in these plots are visualized, where one molecule behaves as a donor (*d*_e_ > *d*_i_), while the other acts as an acceptor (*d*_e_ < *d*_i_). The fingerprint plots highlight the close contacts of specific atom pairs. Thus, selected contributions can account for the stability of the complex crystal structure. The Cl∙∙∙H contacts have the largest contribution in the molecular packing and also have short Cl∙∙∙H distances of 3.49, 3.39, 3.036, 2.87, and 2.81 Å, corresponding to Cl2∙∙∙H1B, Cl1∙∙∙H6B, Cl3∙∙∙H7B, Cl2∙∙∙H6B, and Cl1∙∙∙H7B, respectively. The S∙∙∙H contacts have a relatively strong contribution to the stability of the crystal structure, within the range of 3.22 to 3.43 Å. The proportion of Cl∙∙∙H, H∙∙∙H, S∙∙∙H, and N∙∙∙H interactions comprises 31.9%, 27.2%, 13.5%, and 9.9% of the total Hirshfeld surface, respectively (Figure 6). These are the important interactions which stabilize the crystal structure of the **[Cu_3_(L^a^)_4_(Cl)_6_]** complex.

### 2.4. XPS Studies

**Figure 7:**
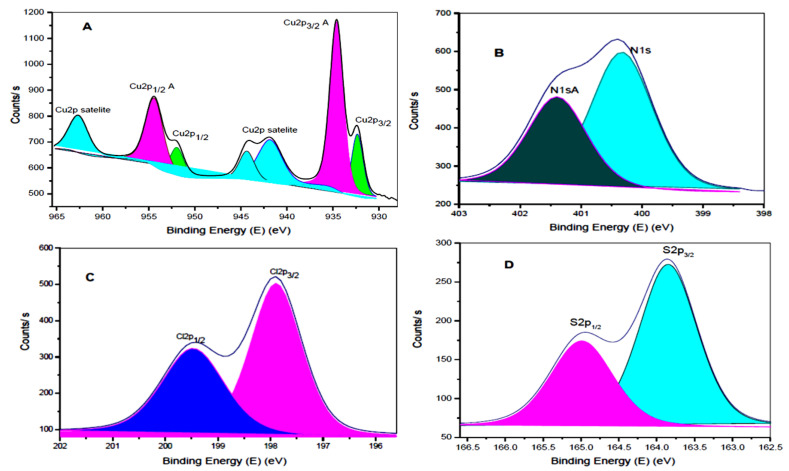
XPS diagram showing binding energy peaks for: (**A**) copper(II) ion in the complex as: Cu2p_1/2_ A, Cu2p_3/2_ A (magenta color), Cu2p_1/2_, Cu2p_3/2_ (green color), and Cu2p satellites (cyan color), (**B**) N1s (cyan color), N1sA (dark cyan color), (**C**) Cl2p_3/2_ (magenta color), Cl2p_1/2_ (blue color), and (**D**) S2p_1/2_ (magenta color), and S2p_3/2_ (cyan color).

X-ray photoelectron spectroscopy is a quantitative measurement for the elemental composition of the material surface, as well as its valence state. Based on single-crystal X-ray structure, the investigated trinuclear copper(II) complex has two distinct coordination environments around copper(II) centers, with coordination numbers 5 and 6. Figure 7 shows the XPS diagram for the **[Cu_3_(L^a^)_4_(Cl)_6_]** complex. The appearance of peaks with binding energies (B.E.) of 932.36 and 934.62 eV corresponding to Cu2p_3/2_ and 951.95, and 954.41 eV corresponding to Cu2p_1/2_, with ∆B.E. of 19.59 and 19.79 eV, respectively, and the three satellites at 941.84, 944.36, and 962.65 eV, confirmed both the oxidation state of copper ions as Cu(II) and the existence of two different environments around copper centers [37,38].

Additionally, the XPS showed two peaks assigned to N1sA and N1s, with B.E. of 401.42 and 400.33 eV, respectively, indicating the existence of two binding types for nitrogen atoms, which are the sp^3^ and sp^2^ (Figure 7B). In addition, the presence of a doublet peak at 197.89 and 199.47 eV, corresponding to Cl2p_1/2_ and Cl2p_3/2_, respectively (∆B.E. = 1.58 eV), indicated its coordinating behavior (Figure 7C) [39,40]. The detailed XPS spectrum showed the two sulfur peaks S2p_3/2_ and S2p_1/2_ [41] at 163.86 and 164.99 eV, corresponding to S2p_3/2_ and S2p_1/2_, respectively. The small value of ∆B.E. (1.13 eV) indicates a non-coordinating S-atom.

### 2.5. Biological Studies

#### 2.5.1. Antimicrobial Activity

The bio-activities of the **[Cu_3_(L^a^)_4_(Cl)_6_]** complex as an antimicrobial agent against Gram-positive bacteria (*S. aureus* and *B. subtilis*), Gram-negative bacteria (*E. coli* and *P. vulgaris*), and the injurious fungi (*A. fumigatus* and *C. Albicans*) were examined and compared with the antibacterial and antifungal control, gentamycin and ketoconazole, respectively. Table 4 summarizes the zone of inhibitions for the complex. The inhibition zone diameters range from 15–20 mm and 13–30 mm against fungi and bacteria, respectively. Generally, the **[Cu_3_(L^a^)_4_(Cl)_6_]** complex has higher activity against Gram-positive bacteria than Gram-negative bacteria. The complex has higher activity against the Gram-positive bacteria *B. subtilis* (30 mm) than *S. aureus* (14 mm). Interestingly, the Cu(II) complex also has a higher activity against *B. subtilis* than the antibacterial control, gentamycin, and equal activity against *C. albicans* when compared with the control compound, ketoconazole. In addition, the antibacterial activity of the Cu(II) complex against Gram-negative bacteria (*E. coli* and *P. vulgaris*) is relatively lower than for the control, gentamycin.

Moreover, the minimum inhibitory concentrations (MIC) in micrograms/mL were determined and depicted in Table 5. The Cu(II) complex showed equal MIC values against *C. albicans* compared to ketoconazole. For the antibacterial activity, the best MIC value for the complex is found against *B. subtilis* (9.7 µg/mL).

#### 2.5.2. Antioxidant Activity

**Figure 8:**
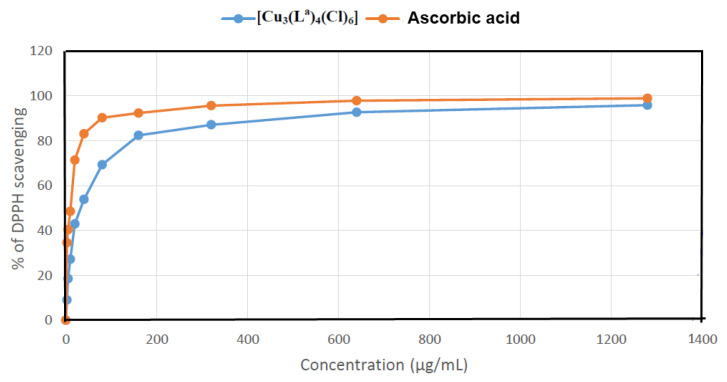
The antioxidant activity of the Cu(II) complex using DPPH assay.

The antioxidant activity of the **[Cu_3_(L^a^)_4_(Cl)_6_]** complex was determined using the DPPH (2,2-diphenyl-1-picryl-hydrazyl-hydrate) method (Figure 8). The percentage of DPPH was determined to be 95.84 at 1280 (µg/mL). Additionally, the IC_50_ value of the **[Cu_3_(L^a^)_4_(Cl)_6_]** complex was determined to be 32.95 ± 1.43 (µg/mL). Under the same experimental circumstance, the reference ascorbic acid has IC_50_ of 10.62 ± 0.84 µg/mL. Thus, the Cu(II) complex showed a relatively reasonable antioxidant activity compared to the reference compound.

#### 2.5.3. Cytotoxic Activity against Colon Carcinoma (HCT-116 Cell)

**Figure 9:**
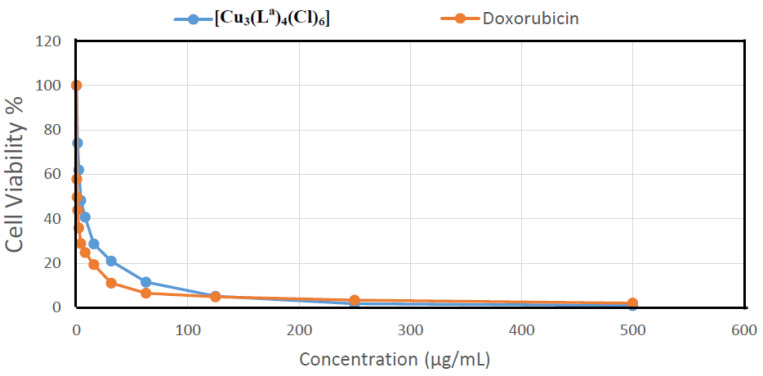
Cytotoxic activity of the **[Cu_3_(L^a^)_4_(Cl)_6_]** complex versus colon carcinoma (HCT-116 cell line).

The **[Cu_3_(L^a^)_4_(Cl)_6_]** complex was tested for its in vitro cytotoxicity against colon carcinoma (HCT-116 cell line). The results of the cytotoxicity experiments are presented graphically in Figure 9. These results showed an extraordinary cytotoxicity against the examined cell line (HCT-116). The percentage of the cell viability is only 0.93 µg/mL at 500 µg/mL, while the IC_50_ value is 3.75 ± 0.43 µg/mL. For doxorubicin as a positive control and under the same experimental conditions, the IC_50_ value is 0.49 ± 0.07 µg/mL. Consequently, this Cu(II) complex has a very promising inhibitory activity against colon carcinoma.

## 3. Experimental

### 3.1. Materials

All chemicals were purchased from Aldrich chemical company and were used without further purifications.

### 3.2. Instrumentations

Details of the instrumentations are described in the Supplementary Material.

### 3.3. Synthesis of 2-(((1-(4,5-Dihydrothiazol-2-yl)ethylidene)hydrazono)methyl)phenol; **L**

To a solution of 1-(4,5-dihydrothiazol-2-yl)ethan-1-one (20 mmol, 2.58 g) in 50 mL ethanol, 1.5 mL of hydrazine hydrate (40 mmol, 50–60%) was added dropwisely, and then the reaction mixture was sonicated for 60 min at 60 °C. The solvent was removed under vacuum, and excess ether was added to afford 2-(1-hydrazanoethyl)dihydrothiazole as a white solid product in higher yield (>90%). The spectral data are in good agreement with the reported data [42,43,44].

Then, 2-(1-hydrazonoethyl)dihydrothiazole (1.143 g, 8 mmol) and salicylaldehyde (0.976 g, 7 mmol) were mixed in 50.0 mL ethanol and 2 drops of AcOH. The reaction mixture was further sonicated for 60 min at 60 °C. Then, the solution was cooled to room temperature, and light-yellow block shaped crystals were formed by slow evaporation of the solution in air after a few days. The yield of the isolated yellow solid was 0.62 g. (90%) [45,46,47].

Anal. Calc. for C_12_H_13_N_3_OS: C, 58.28; H, 5.30; N, 16.99 %. Found: C, 58.09; H, 5.24; N, 16.83 %. ^1^H NMR (400 MHz, DMSO-*d*_6_) δ 8.77 (s, 1H), 7.75 (d, *J* = 7.6 Hz, 1H), 7.41 (t, *J* = 7.0, 6.5 Hz, 1H), 6.99–6.96 (two overlay peaks, 2H), 4.43 (t, *J* = 8.3 Hz, 2H), 3.30 (t, *J* = 8.2 Hz, 2H), 2.33 (s, 3H). ^13^C NMR (101 MHz, DMSO-*d*_6_) δ 169.55 (C=N), 162.81(C=N), 161.85 (C=C-OH), 159.45 (C=N), 132.27(C-Ph), 121.02(C-Ph), 119.01(C-Ph), 117.94(C-Ph), 116.35 (C-Ph), 65.78 (CH_2_N-thiazole ring), 32.17 (CH_2_S), 14.43 (CH_3_).

### 3.4. Synthesis of **[Cu_3_(L^a^)_4_(Cl)_6_]** Complex

A 10 mL ethanolic solution of 2-(1-(4,5-dihydrothiazol-2-yl)ethylidene)hydrazone) methyl)phenol **L** (0.099 g, 4 mmol) was added to 10 mL ethanolic of CuCl_2_.2H_2_O (0.0511 g, 3 mmol) at room temperature. The resulting green solution was filtered out and kept, without disturbing, to evaporate slowly at room temperature. After one week, dark green crystals of the complex **[Cu_3_(L^a^)_4_(Cl)_6_]** were obtained and found appropriate for single crystal structure measurement. Anal. Calc. for C_20_H_28_Cl_6_Cu_3_N_12_S_4_ (80% yield): C, 24.81; H, 2.92; Cu, 19.69; N, 17.36; S, 13.25%. Found: C, 24.59; H, 2.81; Cu, 19.46; N, 17.20; S, 13.09%. IR (KBr, cm^−1^): 1614, 1558, 1427, 1222.

### 3.5. X-ray Structure Determination

Details of the single crystal structure determination are are described in the Supplementary Material [48,49,50].

### 3.6. Biolological Studies

The biological antimicrobial, anticancer and antioxidant activities of the Cu(II) complex were studied. Details of the biological analysis are described in the Supplementary Materials [51, 52, 53].

## 4. Conclusions

A novel **[Cu_3_(L^a^)_4_(Cl)_6_]** complex was synthesized by the reaction of 2-((E)-(((E)-1-(4,5-dihydrothiazol-2-yl)ethylidene)hydrazone)methyl)phenol **L** with CuCl_2_.2H_2_O in Ethanol. This reaction occurs through a Cu(II) mediated hydrolysis of **L,** followed by cyclization to afford the 3-methyl-5,6-dihydrothiazolo[3,2-c][1,2,3]triazole **L^a^**, which undergoes complexation with Cu(II). The structural aspects of the trinuclear **[Cu_3_(L^a^)_4_(Cl)_6_]** complex were determined using single crystal X-ray diffraction, Hirshfeld surface analysis, and XPS. The complex consists of two crystallographically independent **L^a^** ligand units, with one of them acting as a terminal monodentate ligand coordinating only the Cu(2) metal sites, and the other **L^a^** unit acting as a connector between the two Cu(1) and Cu(2) sites. Using Hirshfeld analysis, the Cl∙∙∙H and H∙∙∙H intermolecular contacts are the most prevailing interactions. XPS analysis confirmed the divalent oxidation state of the copper ion. The **[Cu_3_(L^a^)_4_(Cl)_6_]** complex has higher activity against Gram-positive bacteria than Gram-negative bacteria, especially against *B. subtilis.* Additionally, it showed promising antifungal activity against *C. albicans*. Interestingly, the **[Cu_3_(L^a^)_4_(Cl)_6_]** complex has an unexpected cytotoxic activity against the HCT-116 cell line (IC_50_ =3.75 ± 0.43 µg/mL).

## Figures and Tables

**Table 1:** Refinement details of the trinuclear **[Cu_3_(L^a^)_4_(Cl)_6_]** complex.

CCDC	2179746
Empirical formula	C_20_H_28_Cl_6_Cu_3_N_12_S_4_
Formula weight	968.10 g/mol
Temperature/K	296(2) K
Wavelength	1.54178 Å
Crystal system	Triclinic
Space group	*P-1*
*a*/Å	8.1686(3)
*b*/Å	9.4973(4)
*c*/Å	12.7501(5)
*α*/°	81.796(2)
*β*/°	75.291(2)
*γ*/°	65.096(2)
Volume	867.05(6) Å^3^
Z	1
Density (calculated)	1.854 g/cm^3^
Absorption coefficient	8.952 mm^−1^
F(000)	485
Crystal size	0.07 × 0.11 × 0.17 mm^3^
Theta range for data collection	3.59 to 66.53°
Index ranges	−9 ≤ h ≤ 9, −11 ≤ k ≤ 11, −15 ≤ l ≤ 15
Reflections collected	17364
Independent reflections	3055 [*R(int)* = 0.0539]
Completeness to theta = 66.67°	99.70%
Absorption correction	Multiscan
Max. and min. transmission	0.5730 and 0.3110
Refinement method	Full-matrix least-squares on *F*^2^
Data/restraints/parameters	3055/0/213
Goodness-of-fit on *F*^2^	1.067
Final R indices [I > 2sigma(I)]	*R*_1_ = 0.0412*, wR*_2_ = 0.1123
R indices (all data)	*R*_1_ = 0.0442*, wR*_2_ = 0.1164
Largest diff. peak and hole	1.030 and −0.556

**Table 2:** The important geometric parameters in the **[Cu_3_(L^a^)_4_(Cl)_6_]** complex.

**Bond**	**Distance**	**Bond**	**Distance**
Cu1-Cl1	2.2633(10)	Cu2-Cl2	2.3140(13)
Cu1-Cl2	2.8810(11)	Cu2-Cl3	2.2720(12)
Cu1-N2	2.041(2)	Cu2-N1	2.024(3)
Cu2-Cl1	2.6178(11)	Cu2-N4	1.987(3)
**Bonds**	**Angle**	**Bonds**	**Angle**
Cl1-Cu1-Cl2	85.63(3)	Cl1-Cu2-Cl2	91.05(4)
Cl1-Cu1-Cl2 #	94.37(3)	Cl1-Cu2-N4	105.55(9)
N2 -Cu1-Cl1 #	91.33(9)	Cl2-Cu2-N4	89.72(10)
Cl1-Cu1-N2	88.67(9)	N1 -Cu2-N4	166.56(13)
N2 -Cu1-Cl2 #	96.87(9)	Cl2-Cu2-Cl3	171.35(5)
Cl1-Cu2-Cl3	97.12(4)	Cl3-Cu2-N1	90.63(9)
Cl2-Cu1-Cl2 #	180	Cl1-Cu2-N1	87.53(9)
Cl1-Cu1-Cl1 #	180	Cl2-Cu2-N1	86.88(9)
Cl2-Cu1-N2	83.13(9)	Cl3-Cu2-N4	90.82(9)
N2 -Cu1-N2 #	180		

Symm. Code: ^#^-x,1-y,1-z.

**Table 3:** Hydrogen bond parameters (Å, °) in the **[Cu_3_(L^a^)_4_(Cl)_6_]** complex.

D-H...A	d(D-H)	d(H...A)	d(D...A)	<(DHA)	Symm. Code
C1-H1A...Cl1	0.97	2.72	3.427(5)	130	
C6-H6A...Cl1	0.97	2.74	3.609(5)	150	−x,−y,1 − z
C7-H7B...Cl1	0.97	2.81	3.692(5)	150	1 + x, −1 + y, z

**Table 4:** Zone of inhibition (in mm) for the **[Cu_3_(L^a^)_4_(Cl)_6_]** complex.

Microbe	[Cu_3_(L^a^)_4_(Cl)_6_]	Control
*A. fungimatus*	15	17 ^a^
*C. albicans*	20	20 ^a^
*S. aureus*	14	24 ^b^
*B. subtilis*	30	26 ^b^
*E. coli*	18	30 ^b^
*P. vulgaris*	13	25 ^b^

^a^ ketoconazole; ^b^ gentamycin.

**Table 5:** MIC values (µg/mL) for the **[Cu_3_(L^a^)_4_(Cl)_6_]** complex.

Microbe	[Cu_3_(L^a^)_4_(Cl)_6_]	Control
*A. fumigatus*	1250	125.25 ^a^
*C. albicans*	312.5	312.5 ^a^
*S. aureus*	625	9.7 ^b^
*B. subtilis*	9.7	4.8 ^b^
*E. coli*	312.5	4.8 ^b^
*P. vulgaris*	1250	4.8 ^b^

^a^ ketoconazole; ^b^ gentamycin.

## Data Availability

Not applicable.

## Supplementary Materials

The following supporting information can be downloaded at: https://www.mdpi.com/article/10.3390/molecules27144583/s1, Instrumentations; Biological activity methods; Table S1: Evaluation of Antioxidant Activity using DPPH scavenging assay for **[Cu_3_(L^a^)_4_(Cl)_6_]**; Table S2: Evaluation of cytotoxicity against HCT-116 cell line for **[Cu_3_(L^a^)_4_(Cl)_6_]** and Doxorubicin; Figure S1: FTIR spectra of the ligand **L**; Figure S2: FTIR spectra of the trinuclear **[Cu_3_(L^a^)_4_(Cl)_6_]** complex; Figure S3: ^1^H NMR Spectra of 2-(1-hydrazonoethyl)dihydrothiazole; Figure S4: ^13^C NMR of 2-(1-hydrazonoethyl)dihydrothiazole; Figure S5: ^1^H NMR Spectra of **L**; Figure S6: ^13^C NMR of **L**.

## Sample Availability

Samples of the compounds are not available from the authors.
