# Supplementary material for: Synthesis of a Novel Unexpected Cu(II)–Thiazolidine Complex—X-ray Structure, Hirshfeld Surface Analysis, and Biological Studies
Source: Molecules. 2022 Jul 18;27(14):4583. doi: 10.3390/molecules27144583 (PMC9319961; doi:10.3390/molecules27144583)
Supplement: Supplementary file 1 [file molecules-27-04583-s001.zip › molecules-1799356-supplementary.pdf]

Synthesis of a Novel Unexpected Cu(II)–Thiazolidine Complex—X-ray Structure, Hirshfeld Surface Analysis, and Biological Studies

Mezna Saleh Altowyan [1], Samar M. S. M. Khalil [2], Dhuha Al-Wahaib [3], Assem Barakat [4], Saied M. Soliman [2], Ali Eldissouky Ali [2,*] and Hemmat A. Elbadawy [2,*]

[1] Department of Chemistry, College of Science, Princess Nourah bint Abdulrahman University, P.O. Box 84428, Riyadh 11671, Saudi Arabia; msaltowyan@pnu.edu.sa
[2] Chemistry Department, Faculty of Science, Alexandria University, P.O. Box 426, Alexandria 21321, Egypt; samar.mohamed_pg@alexu.edu.eg (S.M.S.M.K.); saied1soliman@yahoo.com (S.M.S.)
[3] Chemistry Department, Faculty of Science, Kuwait University, 13060, Kuwait; d.alwaheeb@ku.edu.kw
[4] Department of Chemistry, College of Science, King Saud University, P.O. Box 2455, Riyadh 11451, Saudi Arabia; ambarakat@ksu.edu.sa
* Correspondence: alieldissouky@alexu.edu.eg (A.E.A.); hemmatabdelfattah@alexu.edu.eg (H.A.E.)

Instrumentations

Elemental analysis was carried out using Perkin-Elmer 2400 Elemental Analyzer; CHN mode. Cu content was determined using Shimadzu atomic absorption spectrophotometer (AA-7000 series, Shimadzu, Ltd., Japan). The FTIR spectra were recorded on a Perkin-Elmer Frontier spectrophotometer as KBr disks. Magnetic Resonance (NMR) spectra were recorded on JEOL-400 MHz MHz spectrometer at ambient temperature in DMSO-d_6 . The single crystal data collection were made on X8 prospector diffractometer (Bruker) by Cu-Kα radiation. The reflection frames were integrated with the SAINT software package (Bruker) using a narrow-frame algorithm and the structures were solved using the SHELXT-2014 (version 5). The structure refinement was performed by SHELXL - 2017/1. Absorption correction was done by multi-scan method. All non-hydrogen atoms were refined anisotropically and hydrogen atoms were refined using the riding model. X-ray photoelectron spectrometry (XPS) measuremnts were performed on a VG ESCALAB 200 spectrometer utilizing Mg$K\alpha$ radiation 1253.6 eV and operating at 300 W (15 kV, 20 mA). All binding energy values were determined with respect to C 1s line (284.6 eV) obtained from adventitious carbon. Depth profiling was done with an Ar ion gun with 5 kV energy and 1-mA current [S1].

Biological activity methods

Antimicrobial assay

All microbial strains were sensitive (non-Resistant) strains provided from the culture collection of the Regional Center for Mycology and Biotechnology (RCMB), Al-Azhar University, Cairo, Egypt. The antimicrobial profile was tested against Gram-positive bacterial species (*Staphylococcus aureus* and *Bacillus subtilis*), Gram-negative bacterial species (*Escherichia coli, Proteus vulgaris*), as well

as against fungi including one filamentous fungus (*Aspergillus fumigatus*) and one yeast species (*Candida albicans*) using the well diffusion method. Briefly, 100 μL of the test bacteria/fungi were grown in 10 mL of fresh media until they reached a count of approximately 10^8 cells/ml for bacteria or 10^5 cells/mL for fungi. One hundred μL of microbial suspension was spread onto agar plates corresponding to the broth in which they were maintained and tested for susceptibility by well diffusion method. One hundred μL of each sample (at 10 mg/mL) was added to each well (6 mm diameter holes cut in the agar gel). The plates were incubated for 24-48 h at 37 °C (for bacteria and yeast) and for 48 h at 28 °C (for filamentous fungi). After incubation, the microorganism's growth was observed. The resulting inhibition zone diameters were measured in millimeters and used as criterion for the antimicrobial activity. Solvent controls (DMSO) were included in every experiment as negative controls. DMSO was used for dissolving the tested compound and showed no inhibition zones, confirming that it has no influence on the growth of the tested microorganisms. Positive controls were also performed using Gentamycin as standard antibacterial drug and Ketoconazole as a standard antifungal drug. All the biologically active samples were subjected to determine the MIC by the broth microdilution method. After incubation, the lowest concentration showing complete inhibition of growth was recorded as the MIC of the respective sample. [S2]

Antioxidant assay

The antioxidant activity of the studied compounds was determined at the Regional Center for Mycology and Biotechnology (RCMB) at Al-Azhar University by the DPPH free radical scavenging assay in triplicate and average values were considered.

DPPH radical scavenging activity

Freshly prepared (0.004%w/v) methanol solution of 2,2-diphenyl-1-picrylhydrazyl (DPPH) radical was prepared and stored at 10 °C in the dark. A methanol solution of the test compound was prepared. A 40 μL aliquot of the methanol solution was added to 3 mL of DPPH solution. Absorbance measurements were recorded immediately with a UV-visible spectrophotometer (Milton Roy, Spectronic 1201). The decrease in absorbance at 515 nm was determined continuously, with data being recorded at 1 min intervals until the absorbance stabilized (16 min). The absorbance of the DPPH radical without antioxidant (control) and the reference compound ascorbic acid were also measured. All the determinations were performed in three replicates and averaged. The percentage inhibition (PI) of the DPPH radical was calculated according to the formula: PI = [{(*A*C- *A*T)/ *A*C} x 100] (1)

Where *A*C = Absorbance of the control at t = 0 min and *A*T = absorbance of the sample + DPPH at t = 16 min.

The 50% inhibitory concentration (IC_{50}), the concentration required to inhibit DPPH radical by 50%, was estimated from graphic plots of the dose-response curve. [S3]

Evaluation of cytotoxic effects against HCT-116 cell line

Mammalian HCT-116 cell line (*colon carcinoma* Carcinoma) cells was obtained from the American Type Culture Collection (ATCC, Rockville, MD, USA).

Chemicals Used: Dimethyl sulfoxide (DMSO), MTT and trypan blue dye was purchased from Sigma (St. Louis, MO, USA). Fetal Bovine serum, RPMI-1640, HEPES buffer solution, L-glutamine, gentamycin and 0.25% Trypsin-EDTA were purchased from Lonza (Belgium).

Cell line propagation

The cells were grown on RPMI-1640 medium supplemented with 10% inactivated fetal calf serum and 50μg/mL of Gentamycin. The cells were maintained at 37ºC in a humidified atmosphere with 5% CO_2 and were sub-cultured two to three times a week.

Cytotoxicity evaluation using viability assay

For antitumor assays, the tumor cell lines were suspended in medium at a concentration 5×10^4 cell/well in Corning® 96-well tissue culture plates, then incubated for 24 h. The tested compounds were then added into 96-well plates (three replicates) to achieve twelve concentrations for each compound. Six vehicle controls with media or 0.5 % DMSO were run for each 96 well plate as a control. After incubating for 24 h, the numbers of viable cells were determined by the MTT test. Briefly, the media was removed from the 96 well plates and replaced with 100 μL of fresh culture RPMI 1640 medium without phenol red then 10 μL of the 12 mM MTT stock solution (5 mg of MTT in 1 mL of PBS) to each well including the untreated controls. The 96 well plates were then incubated at 37°C and 5% CO_2 for 4 hours. An 85 μL aliquot of the media was removed from the wells, and 50 μL of DMSO was added to each well and mixed thoroughly with the pipette and incubated at 37°C for 10 min. Then, the optical density was measured at 590 nm with the microplate reader (SunRise, TECAN, Inc, USA) to determine the number of viable cells and the percentage of viability was calculated as [(ODt/ODc)]x100% where ODt is the mean optical density of wells treated with the tested sample and ODc is the mean optical density of untreated cells. The relation between surviving cells and drug concentration is plotted to get the survival curve of each tumor cell line after treatment with the specified compound. The 50% inhibitory concentration (IC_{50}), the concentration required to cause toxic effects in 50% of intact cells, was estimated from graphic

plots of the dose-response curve for each conc. using GraphPad Prism software (San Diego, CA, USA). [S4]

Table S1. Evaluation of Antioxidant Activity using DPPH scavenging assay for **$[Cu_3(L^a)_4(Cl)_6]$**

**Sample conc. (µg/mL)**	**% DPPH scavenging**	**S.D (±)**
1280	95.84	0.22
640	92.67	0.35
320	87.13	0.41
160	82.39	0.67
80	69.28	1.46
40	53.85	2.31
20	42.93	2.49
10	27.19	2.03
5	18.52	1.48
2.5	9.04	0.21
0	0	0

Table S2. Evaluation of cytotoxicity against HCT-116 cell line for **[$Cu_3(L^a)_4(Cl)_6$]** and Doxorubicin

Conc. (μg/mL)	[$Cu_3(L^a)_4(Cl)_6$]		Doxorubicin	
	**Viability %**	**S.D. (±)**	**Viability %**	**S.D. (±)**
500	0.93	0.25	2.08	0.20
250	1.84	0.42	3.36	0.61
125	5.12	0.36	4.86	0.44
62.5	11.46	0.82	6.51	0.58
31.25	20.97	1.51	11.04	0.72
15.6	28.65	2.17	19.38	1.09
7.8	40.71	0.93	24.82	1.34
3.9	48.23	1.49	28.86	1.12
2	61.98	2.14	35.86	1.62
1	74.06	0.92	43.89	1.07
0	100	0	100	

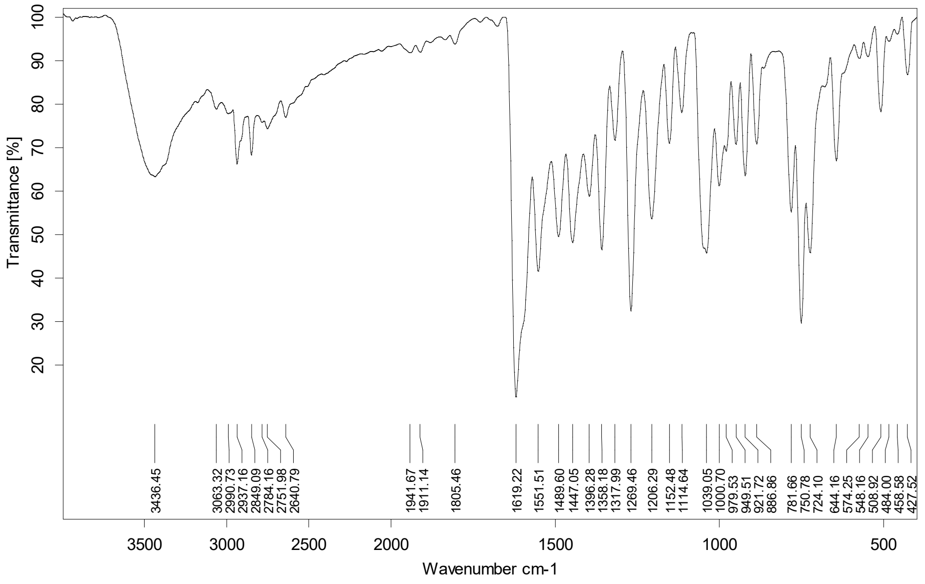

Figure S1. FTIR spectrum of the ligand **L**.

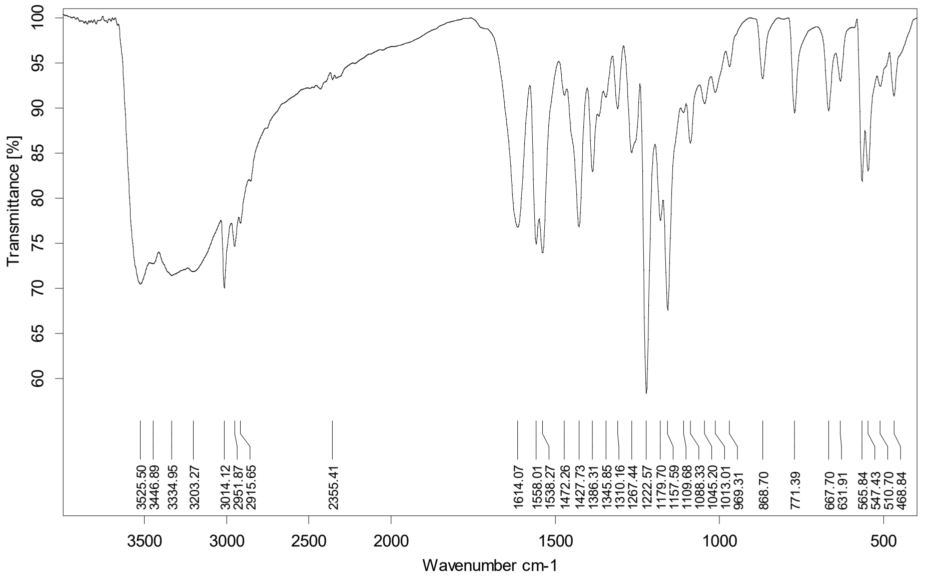

Figure S2. FTIR spectrum of the trinuclear **$[Cu_3(L^a)_4(Cl)_6]$** complex.

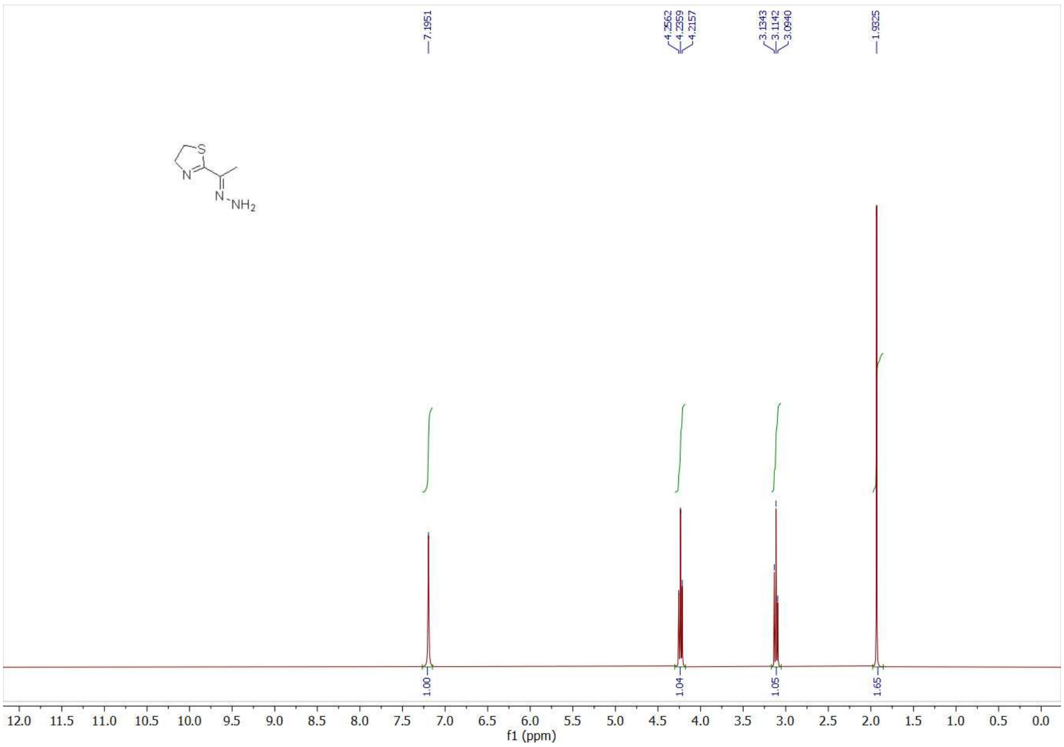

Figure S3. ^{1}H NMR spectrum (400 MHz, DMSO-d_6) of 2-(1-hydrazonoethyl)dihydrothiazole at ambient temperature.

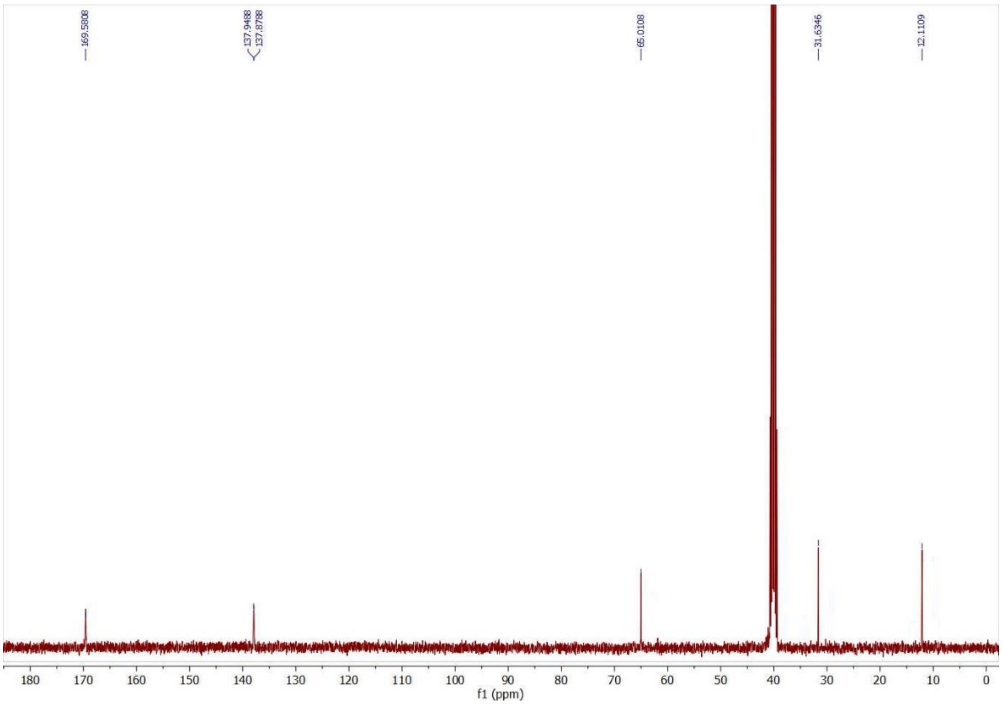

Figure S4. ^{13}C NMR spectrum (100 MHz, DMSO-d_6) of 2-(1-hydrazonoethyl)dihydrothiazole at at ambient temperature.

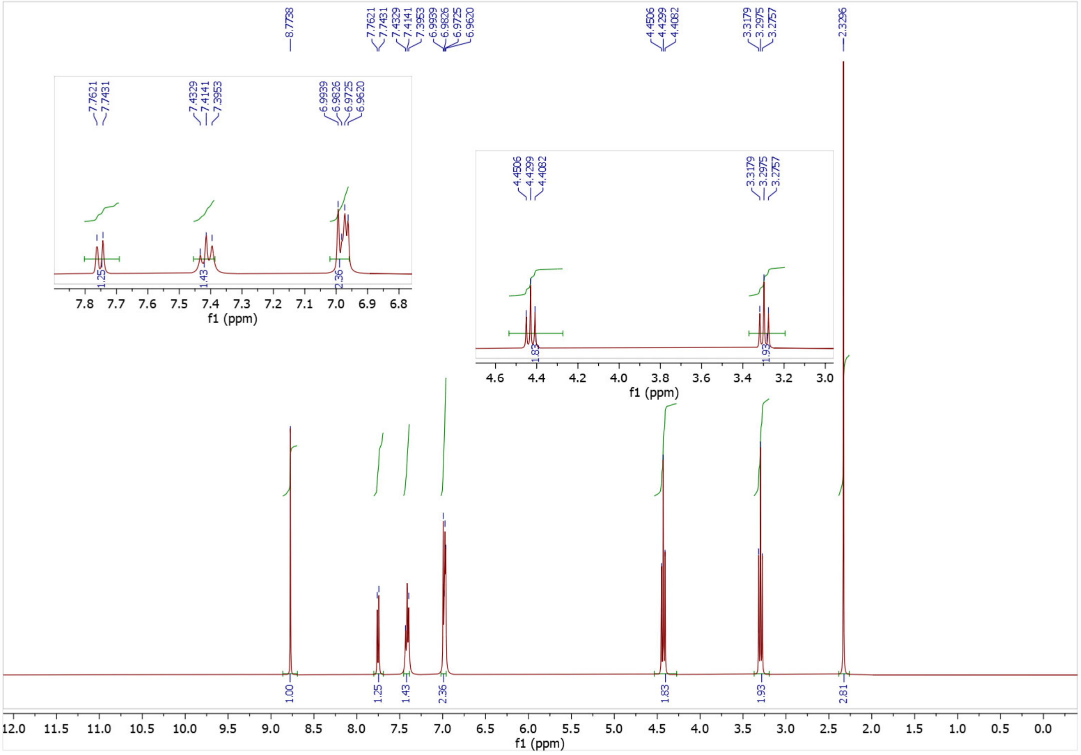

Figure S5. ^1H NMR spectrum (400 MHz, DMSO-d_6) of **L** at ambient temperature.

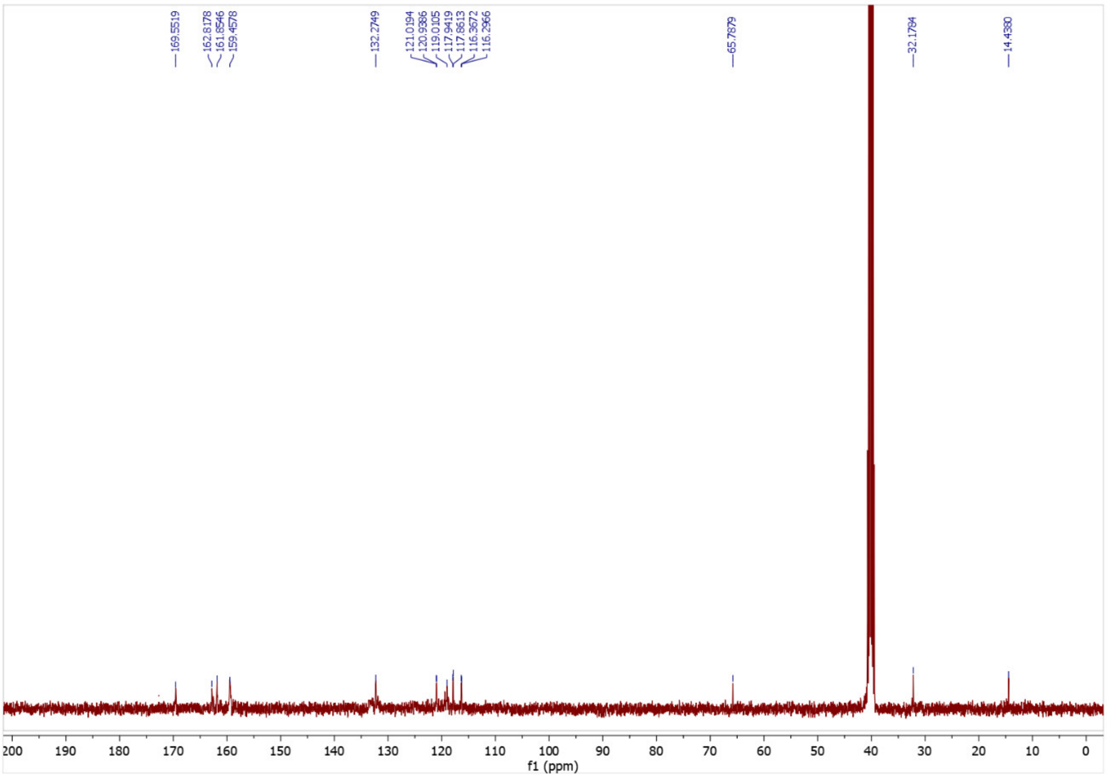

Figure S6. ^{13}C NMR spectrum (100 MHz, DMSO-d_6) of **L** at ambient temperature.
